# Unusual painful pelvic masses: Extramedullary hematopoiesis with extramedullary multiple myeloma

**DOI:** 10.1002/ccr3.8890

**Published:** 2024-05-02

**Authors:** Jennifer Cai, Marian Varda, David Sin, Halline Overby, James J. Yeh

**Affiliations:** ^1^ Department of Pathology Harbor‐UCLA Medical Center Torrance California USA; ^2^ University of California at Irvine Irvine California USA; ^3^ Division of Hematology and Medical Oncology, Department of Medicine Harbor‐UCLA Medical Center Torrance California USA; ^4^ Department of Radiology Harbor‐UCLA Medical Center Torrance California USA; ^5^ Division of Hematology/Oncology, Department of Internal Medicine Martin Luther King Hospital Los Angeles California USA; ^6^ David Geffen School of Medicine at UCLA Los Angeles California USA

**Keywords:** abdominal pain, extramedullary hematopoiesis, extramedullary multiple myeloma, pelvic mass, plasma cell

## Abstract

The coexistence of extramedullary hematopoiesis and extramedullary multiple myeloma can occur and present as painful pelvic masses. In such a case, normal hematopoietic cells may outnumber clonal plasma cells, posing a diagnostic challenge.

## Graphical abstract

Computed tomography‐guided biopsy of the right iliac fossa mass showing extramedullary hematopoiesis with extramedullary multiple myeloma (A, B; yellow arrow: erythroid island; green arrow: myeloid cell; white arrow: megakaryocyte; black arrows: atypical plasma cells with prominent nucleoli; short black arrow: Dutcher body in an atypical plasma cell). CD61 (C) and CD138 (D) immunohistochemistry stains highlight megakaryocytes and plasma cells, respectively.

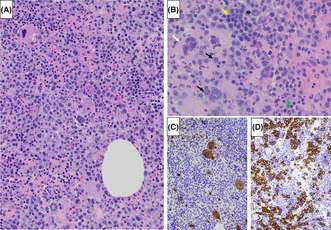

## 1 CASE

**FIGURE 1:**
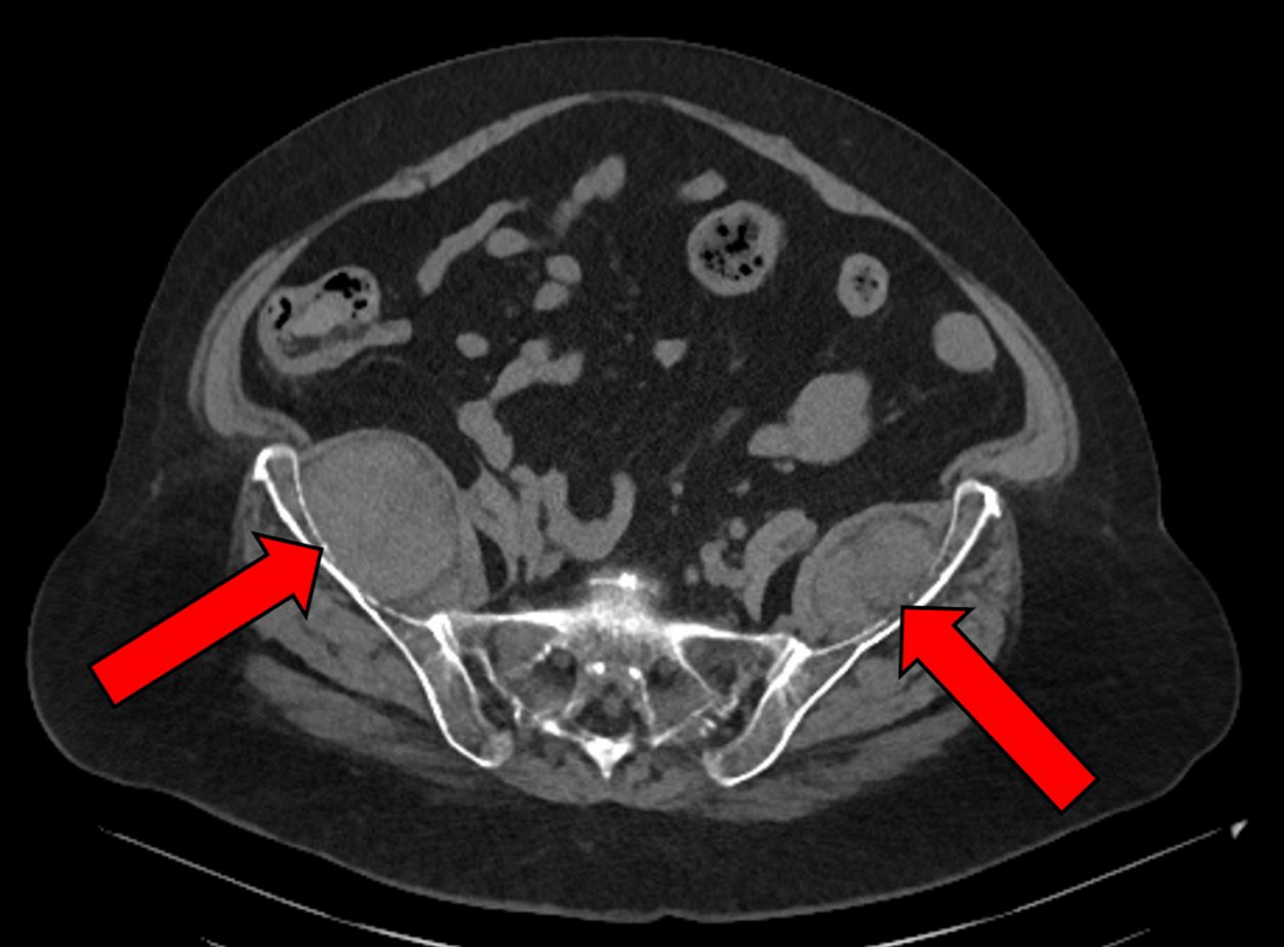
Axial non‐contrast computed tomography at the level of the sacrum demonstrates well‐defined, ovoid soft tissue lesions (red arrows) between the iliac wings and iliacus muscles bilaterally. The lesions are predominantly isoattenuating to the adjacent musculature and contain scattered hypoattenuating foci compatible with macroscopic fat, more appreciable in the left‐sided lesion on this image. There is no definite erosion of the adjacent iliac bone cortices to suggest that the lesions involve the iliac bones. There are intact hypoattenuating fat planes between the lesions and the surrounding iliacus muscles, which suggests that the lesions do not involve the iliacus muscles.

**FIGURE 2:**
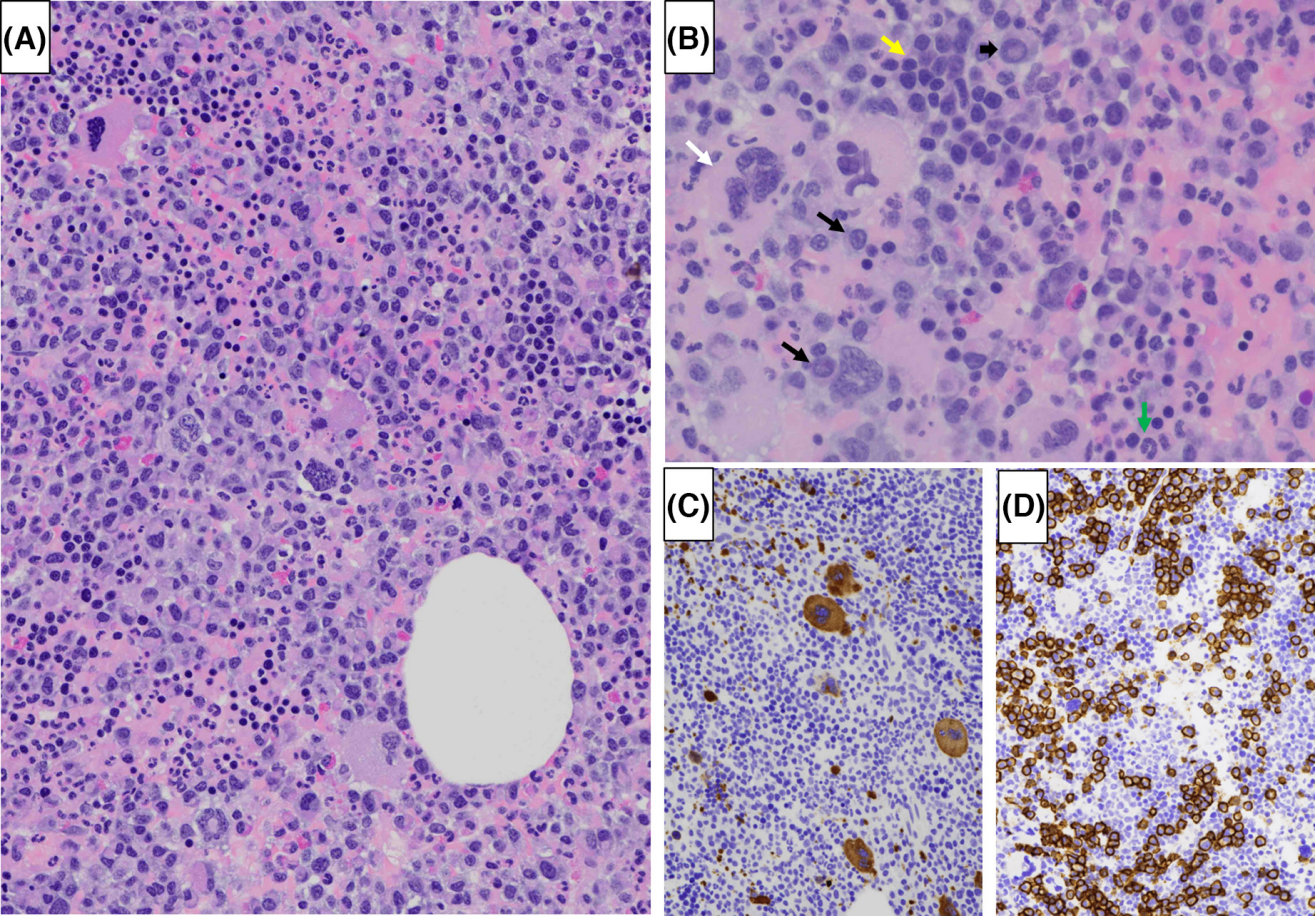
Computed tomography‐guided core biopsy of the right iliac fossa mass shows trilineage (erythroid, myeloid, and megakaryocytic) hematopoiesis with increased atypical plasma cells (A, B; yellow arrow: erythroid island; green arrow: myeloid cell; white arrow: megakaryocyte; black arrows: atypical plasma cells with prominent nucleoli; short black arrow: Dutcher body in an atypical plasma cell). CD61 (C) and CD138 (D) immunohistochemistry stains highlight megakaryocytes and plasma cells, respectively. [A, B: hematoxylin and eosin stain; original magnification, ×200 (A) and × 400 (B). (C, D) immunoperoxidase staining; original magnification, ×200].

A 70‐year‐old female with a past medical history significant for multiple myeloma (diagnosed 3 years before presentation), hypertension, chronic kidney disease, morbid obesity, and cervical spinal stenosis, presented with abdominal pain. She denied a history or family history of hemoglobinopathy, thalassemia, or other hematologic diseases. She had responded well to 8 months of lenalidomide (Revlimid, 25 mg oral daily, Days 1 through 14 of 21‐day cycle), bortezomib (Velcade, 1.3 mg/m^2^, Intravenous, Days 1, 4, 8, 11), and dexamethasone (40 mg Oral Days 1, 8, 15) (RVD) regimen, and maintained a partial remission with weekly Velcade (1.3 mg/m^2^, subcutaneous) and dexamethasone (20 mg, oral). A complete blood count revealed leukopenia (WBC, 4.0 × 10^9^/L), anemia (hemoglobin, 8.9 g/dL), and a normal platelet count. Computed tomography (CT) demonstrated bilateral large pelvic heterogeneous soft tissue masses (right: 6 × 5 cm; left: 5 × 4 cm) near the iliac fossae (Figure 1). CT‐guided core biopsy of the right iliac fossa mass showed trilineage (erythroid, myeloid, and megakaryocytic) hematopoiesis with increased plasma cells (Figure 2A,B). The plasma cells were unevenly distributed (10%–50%) and overall represented approximately 30% of all nucleated cells. Many plasma cells showed atypical features such as enlarged cell sizes, enlarged nuclear sizes, increased nuclear‐to‐cytoplasmic ratios, and distinct/prominent nucleoli. Dutcher bodies were noted in occasional plasma cells. Erythropoiesis, myelopoiesis, and megakaryopoiesis were confirmed by hemoglobin A, CD33, and CD61 (Figure 2C) immunohistochemistry stains. Plasma cells were positive for CD138 (Figure 2D) and immunoglobulin kappa light chain, and negative for immunoglobulin lambda light chain. The findings were consistent with extramedullary hematopoiesis with extramedullary multiple myeloma. The patient received radiation therapy (800 cGy) to her bilateral pelvic masses and resumed weekly Velcade and dexamethasone after a 6‐week break for radiotherapy. A repeated CT 6 months after the radiation therapy showed stable bilateral pelvic heterogenous soft tissue masses (right: 7.5 × 5 cm; left: 5.1 × 3 cm).

## 2 DISCUSSION

Extramedullary hematopoiesis refers to the production of normal blood cells in sites other than the bone marrow and is characterized histologically by increased numbers of erythroid precursors, myeloid precursors, and/or megakaryocytes. It is a rare condition that is typically observed in the liver, spleen, and lymph nodes in patients with hematologic disorders, often without forming tumor‐like masses. It is even more rare for extramedullary hematopoiesis to present as a pelvic mass.^1^ Extramedullary multiple myeloma is a rare form of multiple myeloma and occurs when clonal plasma cells reside outside of the bone marrow and peripheral blood. This is typically associated with high‐risk cytogenetics and poor prognosis.^2^ The pathogenetic mechanisms of extramedullary hematopoiesis or extramedullary multiple myeloma are poorly understood.^2^ It has been suggested that extramedullary multiple myeloma can develop in three ways: (a) direct growth from skeletal tumors following cortical bone disruption; (b) hematogenous spread; or (c) invasive procedures.^3^

This case illustrates an extremely rare, unexpected presentation of extramedullary hematopoiesis with extramedullary multiple myeloma presenting as painful pelvic masses. Consideration of extramedullary multiple myeloma (also called a plasmacytoma) in the setting of extramedullary hematopoiesis in patients with multiple myeloma should be a part of the differential diagnoses of pelvic masses. The mechanism of extramedullary spread of hematopoietic cells and neoplastic plasma cells is unclear in our case, although, given the location, direct growth from bone seems most likely. Extramedullary multiple myeloma typically presents as sheets of clonal plasma cells with minimal bystander hematopoietic cells. In our case, extramedullary multiple myeloma was associated with extramedullary hematopoiesis, and normal hematopoietic cells outnumbered clonal plasma cells, posing a diagnostic challenge.

To our knowledge, the coexistence of extramedullary hematopoiesis and extramedullary multiple myeloma has not been previously described. The biological significance of this association is unknown. Clinicians and pathologists should be aware of this condition, not be confused by bone marrow components in a soft tissue mass and not overlook the neoplastic plasma cells when hematopoietic cells are predominant.

## AUTHOR CONTRIBUTIONS

**Jennifer Cai:** Conceptualization; investigation; methodology; project administration; visualization; writing – original draft; writing – review and editing. **Marian Varda:** Investigation; project administration; resources; writing – review and editing. **David Sin:** Methodology; resources; visualization; writing – review and editing. **Halline Overby:** Investigation; project administration; resources; writing – review and editing. **James J. Yeh:** Investigation; project administration; resources; writing – review and editing.

## FUNDING INFORMATION

None.

## CONFLICT OF INTEREST STATEMENT

None.

## ETHICAL APPROVAL

Ethical review and approval of the study are not applicable in this case.

## CONSENT

Written informed consent was obtained from the patient to publish this report in accordance with the journal's patient consent policy.

## Data Availability

No datasets were generated or analyzed during the current study.
